# Novel coating containing molybdenum oxide nanoparticles to reduce *Staphylococcus aureus* contamination on inanimate surfaces

**DOI:** 10.1371/journal.pone.0213151

**Published:** 2019-03-18

**Authors:** Susana Piçarra, Elizeth Lopes, Pedro L. Almeida, Hermínia de Lencastre, Marta Aires-de-Sousa

**Affiliations:** 1 Centro de Química Estrutural-CQE, DEQ, Instituto Superior Técnico, Universidade de Lisboa, Lisboa, Portugal; 2 Escola Superior de Tecnologia do Barreiro, Instituto Politécnico de Setúbal, Rua Américo da Silva Marinho, Lavradio, Portugal; 3 Laboratory of Molecular Genetics, Instituto de Tecnologia Química e Biológica António Xavier (ITQB), Universidade Nova de Lisboa (UNL), Oeiras, Portugal; 4 CENIMAT/I3N, Departamento de Ciência dos Materiais, Faculdade de Ciências e Tecnologia (FCT), Universidade Nova de Lisboa (UNL), Caparica, Portugal; 5 Área Departamental de Física, Instituto Superior de Engenharia de Lisboa (ISEL) Instituto Politécnico de Lisboa (IPL), Lisboa, Portugal; 6 Laboratory of Microbiology and Infectious Diseases, The Rockefeller University, New York, United States of America; 7 Escola Superior de Saúde da Cruz Vermelha Portuguesa (ESSCVP), Lisboa, Portugal; Institute of Materials Science, GERMANY

## Abstract

We previously synthetized molybdenum oxide (MoO_3_) nanoparticles (NP) and showed their antibacterial activity against a representative collection of the most relevant bacterial species responsible for hospital-acquired infections, including *Staphylococcus aureus*. The aim of the present study was to prepare and characterize a novel coating with these MoO_3_ NP, confirm its mechanical stability, and investigate its biocidal effect to reduce *S*. *aureus* contamination on inanimate surfaces. In addition, the novel MoO_3_ NP coating was compared to a silver (Ag) NP coating synthetized by the same procedure. The MoO_3_ and Ag NP coatings were characterized in terms of their chemical structure by FT-IR, surface morphology by scanning electron microscopy, and mechanical properties by tensile and adhesion tests. The antimicrobial activity of the coatings was tested by following the loss of viability of *S*. *aureus* after 6h, 24h, 48h, and 72h exposure. MoO_3_ and Ag coatings exhibited surfaces of comparable morphologies and both presented elastomeric properties (tensile strength of ~420 kPa, Young’s modulus of ~48 kPa, and maximum elongation of ~12%), and excellent (classification of 5B) adhesion to glass, steel and polystyrene surfaces. The two coatings exhibited a good antibacterial activity (R) against *S*. *aureus* over time (R_MoO3_ = 0.2–0.81; R_Ag_ = 0.61–2.37), although the effect of the Ag NP coating was more pronounced, especially at 72h (R_MoO3_ = 0.81 vs R_Ag_ = 2.37). Noteworthy, contrary to the Ag NP coating, the MoO_3_ NP coating was colourless and transparent, avoiding undesired unaesthetic effects. The synthetized coating with NP of MoO_3_, which has low toxicity to humans, capability of biodegradation, and rapid excretion, can be applied onto most standard materials and therefore is a promising tool to reduce *S*. *aureus* contamination on usual inanimate surfaces found in healthcare and community environments.

## Introduction

*Staphylococcus aureus* remains a leading cause of bacterial infections worldwide, ranging from skin and soft tissue infections to more severe conditions such as bacteremia, meningitis, pneumonia, osteomyelitis, and endocarditis. The high morbidity and mortality associated to *S*. *aureus* infections is due largely to its methicillin resistant form (MRSA), which was historically associated exclusively with hospital-acquired infections, but subsequently spread also to the community posing new threats and challenges.

*S*. *aureus* is able to survive on a variety of environmental surfaces, over a wide range of temperatures, humidity, and exposure to sunlight promoting contamination of inanimate surfaces [1]. Therefore, although transmission of *S*. *aureus* occurs mainly by direct human-to-human skin contact, environmental surfaces represent an important reservoir for dissemination as well.

We have shown that public transportation constitutes an indubitable reservoir of MRSA and a transmission route from the hospital to the community [2, 3]. It is also well known that hospital environmental surfaces represent a source of infection and colonization for patients and healthcare workers. Therefore, efficient measures to reduce *S*. *aureus* environmental surfaces contamination are urgently needed in the healthcare system and in the community.

Nanostructure materials have gained increased attention in the biomedical field due to their antimicrobial activity in low concentrations [4]. Out of the different nanomaterials considered for their antibacterial properties, silver (Ag) nanoparticles (NP) have been extensively studied since they possess remarkable broad-spectrum antimicrobial capacities [5, 6]. However, the high cost of silver and the toxicity of Ag NP limits its applications [7].

The antibacterial action of Ag NP has been mainly attributed to the action of Ag^+^ ions in membrane disruption, generation of reactive oxygen species (ROS) stress and genotoxic damage, which leads to cell death [8]. The ROS production has been recently linked to the interaction of Ag NP with different cellular proteins [9, 10]. However, the bactericidal effect of MoO_3_ NP has been attributed to the release of hydronium ions affecting enzyme activity, protein stability, and structure of nucleic acids, thus killing the bacteria [11]. We have previously synthetized pure molybdenum oxide (MoO_3_) NP, a non-toxic compound, and recently showed their antibacterial activity against a representative collection of the most relevant bacterial species responsible for hospital-acquired infections, including *S*. *aureus* [12].

The aim of the present study was to prepare and characterize a novel coating with these MoO_3_ NP, confirm its mechanical stability, and investigate its biocidal effect to reduce *S*. *aureus* contamination on inanimate surfaces. In addition, the novel MoO_3_ NP coating will be compared to a Ag NP coating synthetized by the same procedure.

## Methods

### Synthesis of MoO_3_ and Ag NP coatings

MoO_3_ NP (Sigma-Aldrich, St. Louis, MO, USA) were prepared following the one-step thermal decomposition approach using ammonium heptamolybdate tetrahydrate [(NH_4_)_6_Mo_7_O_24_.4H_2_O] (Chem-Lab NV, Zedelgem, Belgium) as starting precursor, for 4h at 500°C [12]. Ag NP (purity 99.5%; Sigma-Aldrich, St. Louis, MO, USA) and N1-(3-trimethoxysilylpropyl)diethylenetriamine (SiDETA) (technical grade, Sigma-Aldrich, St. Louis, MO, USA) were used as received.

NP coatings of MoO_3_ and Ag (MoO_3_CT and AgCT) were prepared by dispersing 0.118 mmol of NP (0.085 g for MoO_3_; 0.0637g for Ag) into 50 ml of ethanol (EtOH). After 20 min of sonification, 50 μl of SiDETA was added, during stirring. A coating without NP (CT) was also prepared, directly from a 1.9 mM solution of SiDETA in EtOH.

Films from the three above described coatings (CT, AgCT and MoO_3_CT) were prepared on different substrates (glass, polystyrene and steel AA2024) by dip coating (three dives with ascending/descending movements of 5 s), spraycoating (three sprays of 5 s each), and by brush (painted twice with 15 minutes break between them).

### Morphologic and chemical characterization of MoO_3_ and Ag NP coatings

The morphology of the films deposited by dip coating was assessed by using a scanning electron microscope CrossBeam Workstation (SEM-FIB)–Zeiss Auriga. The SEM images under the in-lens mode were carried out with an acceleration voltage of 2 kV and aperture size of 30 μm. A thin carbon layer (< 20 nm) was deposited on the suspended fibers using a Q150T ES Quorum sputter coater.

Coatings chemical structure was unveiled by FT-IR. The FT-IR data were obtained using an Attenuated Total Reflectance (ATR) sampling accessory (Smart iTR) equipped with a single bounce diamond crystal on a Thermo Nicolet 6700 Spectrometer. The spectra were acquired with a 45° incident angle in the range of 4000−600 cm^−1^ and with a 4 cm^−1^ resolution.

### Tensile and adhesion tests

Mechanical tensile tests were performed on samples from the three coatings. In order to have free standing films to do the tensile tests, the films were allowed to set on a Teflon mould. From the obtained films (with thicknesses of around 50 μm), test specimens were cut with the dimensions of 15x30 mm^2^, leaving after assembly a distance between clamps (l_0_) of 10 mm. The tensile test machine used was Rheometric Scientific (Minimat–Firmware V. 3.1) testing machine. The measurements were carried out at room temperature (25°C). Five successful determinations were used to obtain average values. The stretching rate was 5 mm/min. The stress, σ, was determined in terms of the original cross-sectional area (A_0_), and the strain defined as ε = (l-l_0_)/l_0_, where l and l_0_ are the length at the time of data collection and original length, respectively.

The three produced coatings were tested for their adhesion to different substrates according to the American Society for Testing and Materials Standard D3359-17: “Standard Test Methods for Rating Adhesion by Tape Test” [13]. In the Standard, two testing methods are defined A and B. Test method B is more suitable for use in laboratory environment, having the limitation of the coating thickness not to exceed 125 μm. Three substrates (polystyrene, steel and glass) were used for the adhesion testing coated with CT, MoO_3_CT and AgCT, with a thickness under 100 μm after solvent evaporation. The adhesion tests were performed in triplicate for each sample.

### Antimicrobial activity of the MoO_3_ NP coating

A bacterial suspension (100 μl) of *S*. *aureus* strain ATCC25923 adjusted to 0.5 McFarland was spread on sterilized microscope glasses covered with the coating with MoO_3_ NP and on glasses with the coating without NP (control). After drying the glasses were scrubbed with a swab moistened in sterile water that was subsequently inserted into 1 ml H_2_O and vortexed for 1 minute. Serial dilutions were inoculated onto Tryptic Soy Agar (TSA, Becton, Dickinson & Co, New Jersey, USA) plates which were incubated at 37°C overnight (ON). The same procedure was repeated for 6 h, 24 h, 48 h and 72 h.

The antimicrobial activity (R) of the MoO_3_ NP coating was calculated using the following equation:

$$R={(A}_{0}-A_{t})-{(C}_{0}-C_{t})$$ (1)

where A and C are the common logarithm of the number of viable cells recovered from the surfaces covered with the coating with NP (A) and from the surfaces covered with the coating without NP (C-control) immediately after inoculation (A0 and C0) and after each incubation time (At and Ct).

The experiments were performed in triplicate and the antimicrobial activity was calculated with the average colony forming units (CFU) counts.

### Comparison of the antimicrobial activity of the MoO_3_ and Ag NP coatings

Considering the well-known antibacterial properties of silver, we additionally evaluated the antimicrobial activity of the synthetized Ag NP coating against *S*. *aureus* for comparison of the biocidal effect of the MoO_3_ NP coating. We performed the assay as above, with microscope glasses covered with coatings with Ag NP using three different concentrations (Ag_100%_—same NP concentration used for the MoO_3_ NP coating [2.36 mM], Ag_50%_—half NP concentration [1.18 mM], and Ag_25%_—one quarter NP concentration [0.59 mM]). The antimicrobial activity was calculated as above.

## Results and discussion

### Synthesis of MoO_3_ and Ag NP coatings

**Fig 1:**
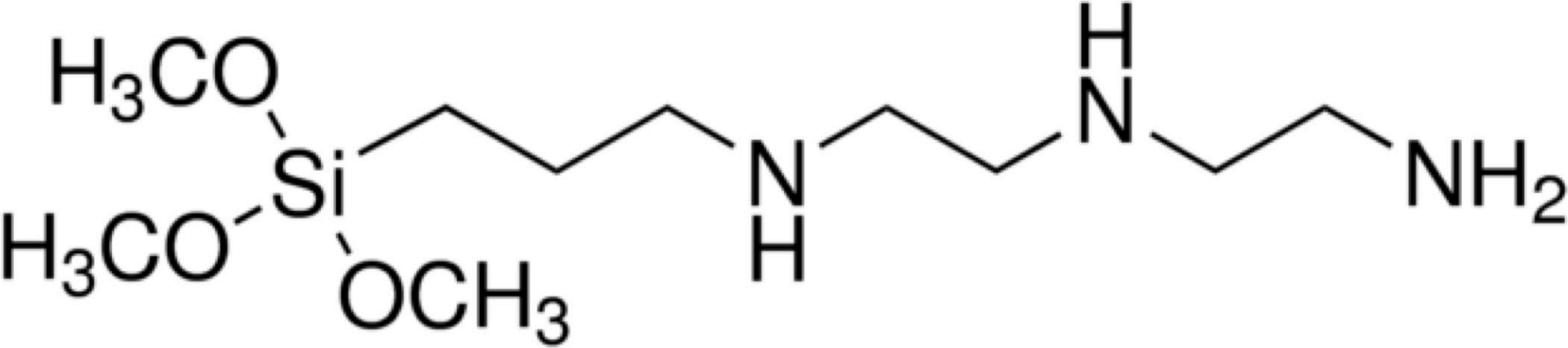
Molecular structure of N1-(3-trimethoxysilylpropyl)diethylenetriamine (SiDETA).

SiDETA is an ORMOSIL compound composed by a head containing three alkoxysilyl groups and by an organic tail containing three amino groups (Fig 1). In the presence of water (from EtOH solution and from atmospheric moisture), alkoxysylane groups from the SiDETA *head* suffer hydrolysis and condensation through the very well-known sol-gel process, originating a silica network that constitutes the coating (and water and methanol as byproducts) [14, 15]. This process is, however, very much dependent on the reactional medium conditions (temperature, moisture, pH, etc.) and the introduction of Ag or MoO_3_ NP might result in networks with distinct structures, originating coatings with distinct properties. All synthesis were made under atmospheric pressure and at 20±5°C. SiDETA pH was 10.88 ± 0.49, which remained stable after the induction of NP.

### Characterization of MoO_3_ and Ag NP coatings

**Fig 2:**
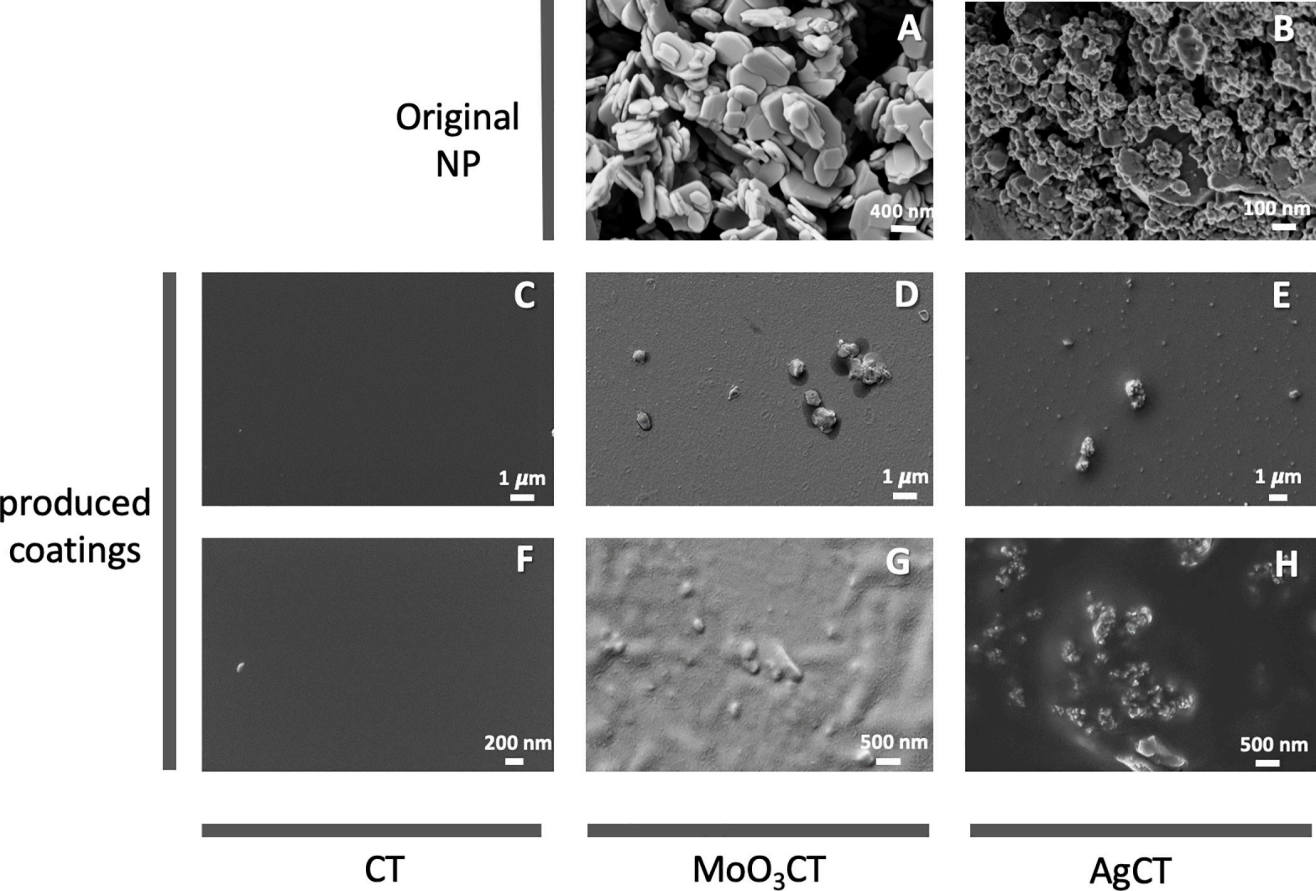
SEM-FIB images of the MoO_3_ and Ag NP (A and B, respectively) and the produced coatings; CT (C and F), MoO_3_CT (D and G), and AgCT (E and H).

The SEM-FIB images of MoO_3_ and Ag NP showed that both NP present 2D plate-like morphologies (Ag NP showing rougher surfaces than MoO_3_ NP) with average dimensions of 200–800 nm and 50–200 nm, respectively (Fig 2A and 2B). SEM-FIB images of the produced SiDETA coatings showed that while the CT surfaces are smooth (Fig 2C and 2F), MoO_3_CT (Fig 2D and 2G) and AgCT (Fig 2F and 2H) surfaces are rough, showing aggregates of NP of similar dimensions (0.5 to 1.0 μm), independently of the individual dimensions of the MoO_3_ and Ag NP.

**Fig 3:**
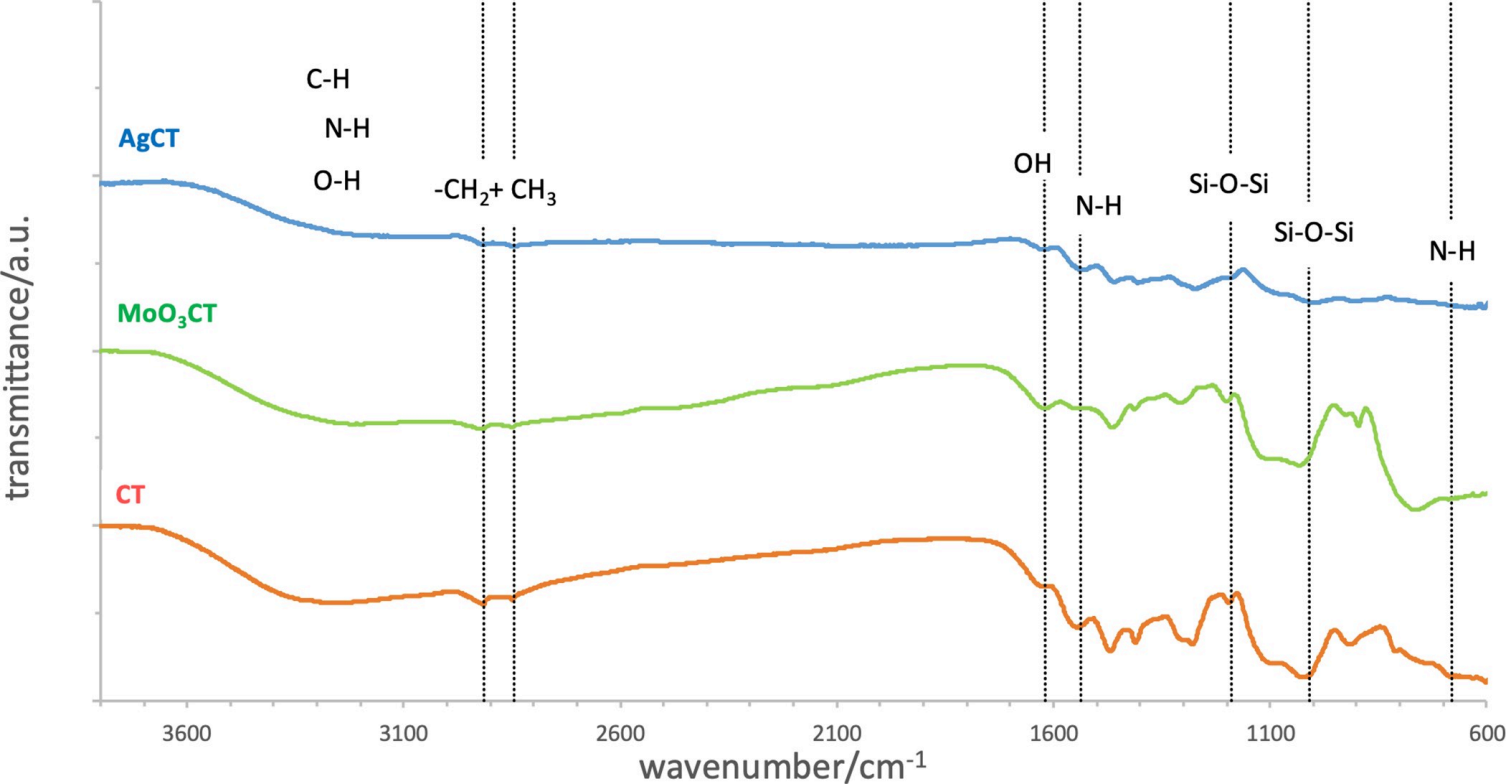
FT-IR spectra of the three coatings (CT, MoO_3_CT, and AgCT).

The FT-IR spectra of the three coatings (CT, MoO_3_CT, and AgCT) are, however, quite similar (Fig 3), all spectra revealing the characteristic patterns of a silica network and amine and methylene groups.

From the FT-IR spectra it is possible to conclude that independently of the presence of MoO_3_ and Ag NP, the alkoxysilane groups of SiDETA *head* had suffered similar sol-gel reactions and formed networks with similar chemical bounds. We could also observe that the organic amine groups containing *tail* remained in the formed network, as expected. The presence of the organic *tail* should be responsible for introducing elasticity into the resulting coating material and the amino groups to increase the coating adhesion properties.

Therefore, the three produced coatings (CT, MoO_3_CT and AgCT) were tested for their adhesion to different substrates (polystyrene, steel and glass) [13]. All samples obtained an adhesion classification of *5B* according to the standard classification which means that the edges of the cuts are completely smooth; none of the squares of the cut lattice was detached. These adhesion assays proved that the coatings (with and without NP) have a strong adhesion to the tested substrates and therefore are suitable to coat most standard materials and therefore suitable for usual surfaces found in healthcare and community environments.

Mechanical tensile tests were additionally performed on samples from the three coatings. The tensile stress–strain curves, for the range of weight ratios (HPC/BDI) studied, did not exhibit a yield point. The tensile strength, the elongation, and the Young’s modulus did not change significantly between samples indicating that the presence of MoO_3_ and Ag nanoparticles does not affect the mechanical behavior of the formed networks that constitute the coatings. These coatings mechanically behave as rigid elastomers presenting a tensile strength of around 420 kPa, a Young’s modulus of around 48 kPa and a maximum elongation of around 12%. The determined mechanical properties clearly state that these materials are suitable for this application [16], presenting mechanical robustness that allows the coating to resist to its practical usage.

**Fig 4:**
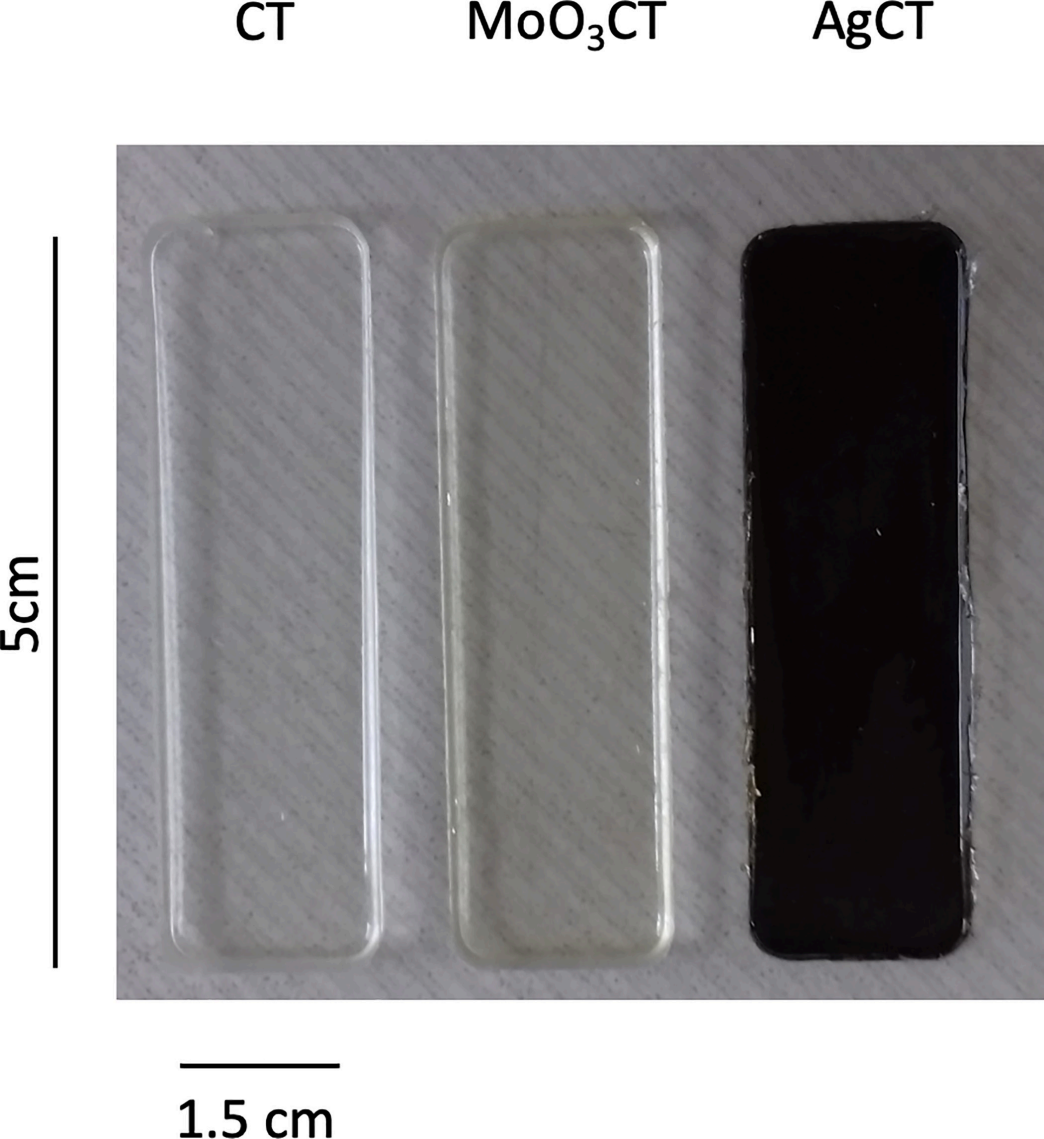
Digital photographs of microscope glasses covered with the three coatings (CT, MoO_3_CT, and AgCT).

While Ag NP containing coating is visually aesthetical (black and opaque) which limits several applications, MoO_3_ NP are colorless and transparent, being suitable to be applied on a diversity of surfaces in both clinical and community settings (Fig 4).

### Antimicrobial activity of the MoO_3_ and Ag NP coatings

**Fig 5:**
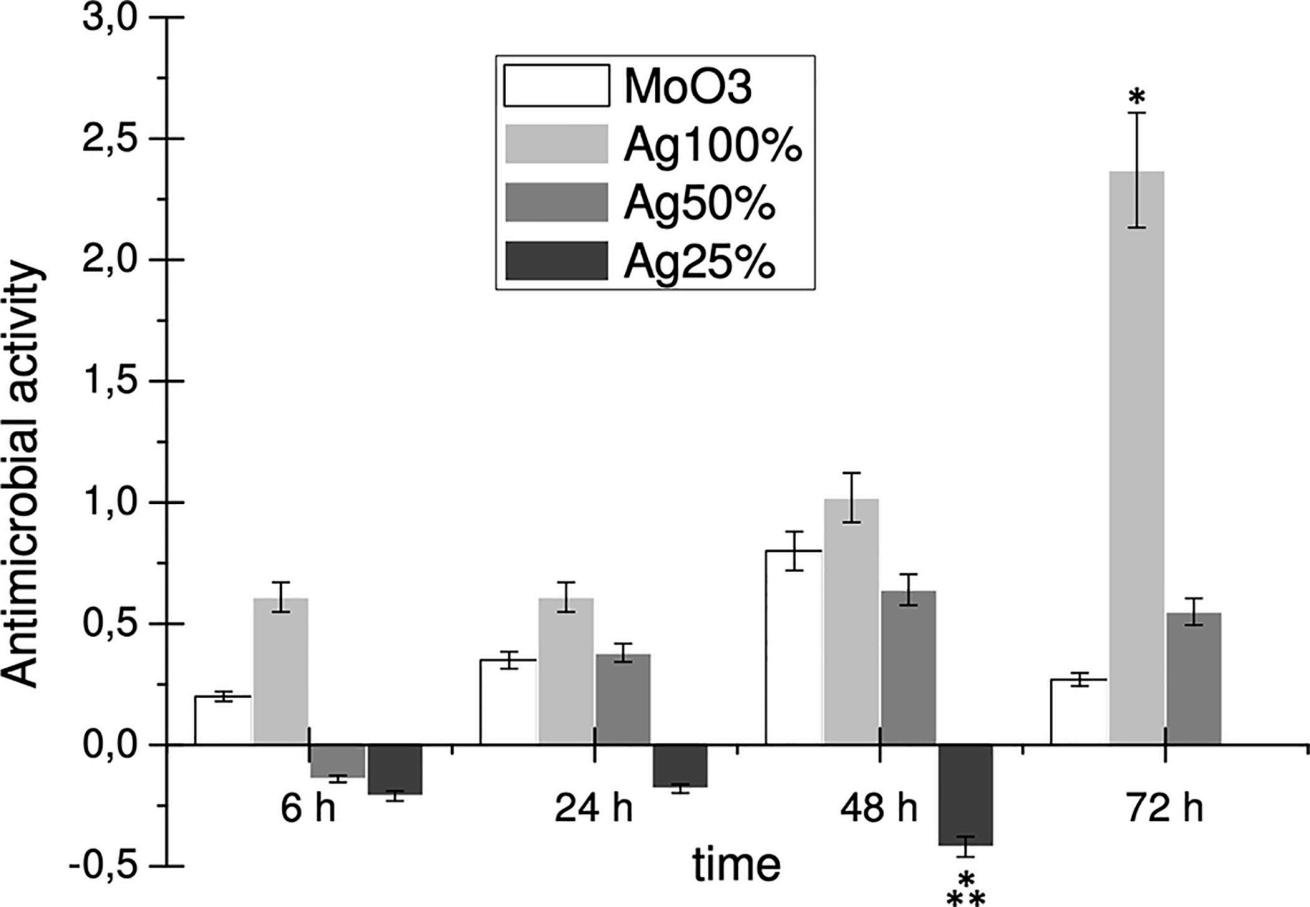
Antimicrobial activity of MoO_3_ and Ag NP coatings against *S*. *aureus* strain ATCC25923. * and ** denote significant change (p< 0.05) compared to MoO_3_ and Ag100, respectively.

The results demonstrated that the synthetized MoO_3_ NP coating exhibited antibacterial activity against *S*. *aureus*, in a time-dependent way until 48 h of contact time (Fig 5). Noteworthy, the biocidal effect of the MoO_3_ NP coating was also verified after 28 days indicating its long-term stability on glass surfaces (results not shown).

**Fig 6:**
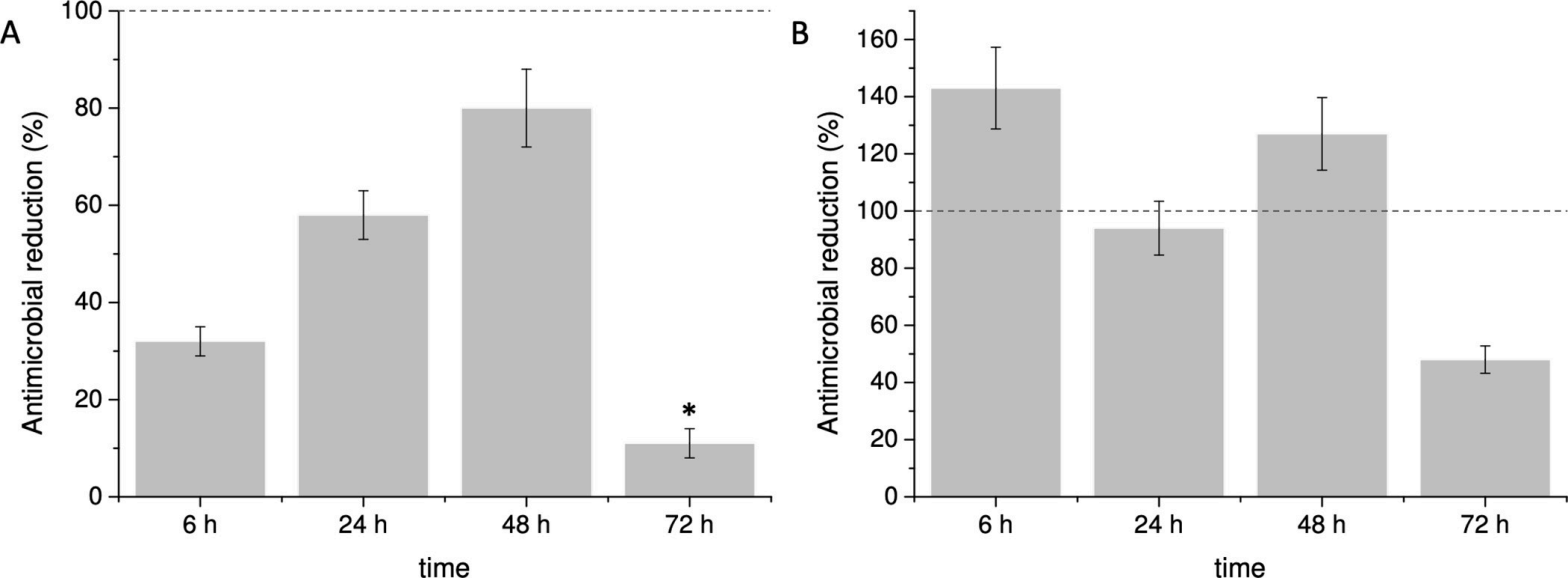
Comparison of the antibacterial effect of the MoO_3_ NP coating with two Ag NP coatings: A) Ag_100%_—same NP concentration used for the MoO_3_ NP coating; B) Ag_50%_—half NP concentration. * denotes significant change (p< 0.05).

Considering the well-known antibacterial properties of silver, we prepared a coating containing Ag NP as a positive control, using the same procedure and the same amount of NP (Ag_100%_). When comparing the antibacterial activity of the MoO_3_ and the Ag_100%_ NP coatings towards *S*. *aureus*, under the same conditions, the former showed a lower antibacterial activity (Fig 5). Nevertheless, the MoO_3_ NP coating reached 58% and 80% of the biocidal effect of the Ag_100%_ NP coating at 24 h and 48 h, respectively (Fig 6A). Remarkably, the antimicrobial efficacy was slower for the MoO_3_ NP coating, since after 6 h of contact it reached just one third of the Ag_100%_ NP effect and lasted for a shorter period of time showing only 11% of the effect of the Ag_100%_ NP at 72 h (Fig 6A).

Decreasing the Ag NP concentration on the coating resulted in a decreased antimicrobial activity, and when the NP concentration was reduced to 25%, the biocidal effect was totally abolished (Fig 5). The results showed that the MoO_3_ coating was at least as efficient as the Ag_50%_ NP coating until 48 h of contact, showing a much faster biocidal effect for 6 h and even an increased efficacy at 48 h (Fig 6B).

**Fig 7:**
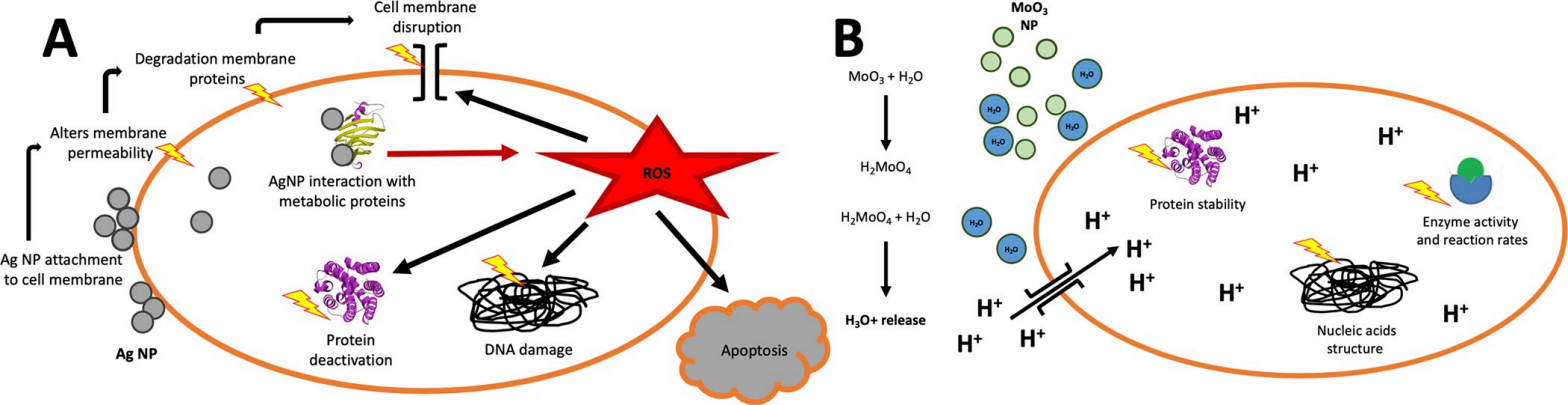
Schematic diagram of the mechanism of antimicrobial action of Ag (A) and MoO_3_ (B) NP.

The differences observed in the antimicrobial effectivity of the MoO_3_ and Ag coatings might be due to the different modes of action of the NP (Fig 7). The Ag NP act as an antimicrobial by a combination of several mechanisms: (i) adhesion to the bacterial cell wall, followed by penetration, causing structural alterations in the cell membrane and subsequent death of the bacteria, (ii) formation of free radicals by the Ag NP which damage the cell membrane leading to cell death, (iii) release of silver ions (Ag^+^) which inactivate several vital enzymes and inhibit numerous functions in the cell, and (iv) generation of reactive oxygen species (ROS), that damage DNA and mitochondria, cause oxidation of proteins and lipids, cell membrane disruption and activate apoptosis [17–19]. In contrast, the antibacterial effect of the MoO_3_ NP is due to the creation of an acidic environment by conversion of MoO_3_ into molybdic acid (H_2_MoO_4_) and subsequent release of H_3_O^+^ ions, which requires the presence of water. The hydronium ions diffuse into the cell membranes affecting enzyme activity and reaction rates, protein stability, and structure of nucleic acids, thus killing the bacteria [11]. Therefore, when embedding the MoO_3_ NP in the coating, the interaction with water is restricted to the contact time with the aqueous bacterial suspension, which might explain the pronounced reduction in the antibacterial activity at 72h (the surface has considerably dried) as well as a less pronounced antimicrobial effect compared to the NP in an aqueous solution, as previously shown [12].

An effective antimicrobial coating must include several characteristics: (i) be able to reduce the pathogenic population on a surface; (ii) be stable (mechanically and chemically); (iii) minimize toxicological effects and risks of emergence of antimicrobial resistance; (iv) be affordable and easily implemented [20]. The MoO_3_ NP coating synthetized in the current study is mechanically and chemically stable, presented rigid elastomeric properties, exceptional adhesion to glass, steel and polystyrene surfaces, and has proved to be able to control a *S*. *aureus* population on a surface. Moreover, molybdenum has low toxicity to humans, capability of biodegradation, and rapid excretion [21], and to the best of our knowledge bacterial resistance to MoO_3_ NP has not been described so far. In addition, Ag NP containing coating is visually unaesthetic which limits several applications, while MoO_3_ is colorless and transparent.

In conclusion, we synthetized a novel MoO_3_ NP coating using an easy and timesaving protocol. The characterization of the coating showed it can be applied onto most standard materials, it has antimicrobial activity against *S*. *aureus*, and therefore is suitable to reduce bacterial contamination on usual surfaces found in healthcare and community environments.
